# Observations on the Deranged Manifestations of the Mind, or Insanity

**Published:** 1818-05

**Authors:** 


*Observations on the deranged Manifestations of the Mind, or Insanity.* By J. G. Spurzheim, M. D. Licentiate of the College of Physicians of London, &c. With Plates. Large 8vo. pp. 312. London, 1817.

On a late occasion,* we were amongst the foremost of our fellow-Journalists to vindicate the honest claims of Dr. Spurzheim to superior eminence in anatomical knowledge of the brain, and to reprobate the disgraceful persecution which was set on foot against him in a sister-metropolis; a persecution that has scarcely been paralelled since the times of Calvin and Servetus, for downright inveteracy of hate and unfairness of purpose. We own our sympathetic feelings were greatly roused, to think that a man of unquestionable talents, and as unquestionable rectitude of intention, should be assailed by the flippant dogmatism, subtlety, or raillery, of practised hyper-critics, lawyers, and ladies,—whether in lecture-

---

* *See* Med. Chir. Journ. Vol. iv. p. 53.

rooms, reading-rooms, or drawing-rooms,—whether in public or private; and that a digested plan of contumely and intentional misrepresentation should be perseveringly followed out against him by a trained band of myrmidons and retainers, of whom it might be truly said, as of the ten virgins, that "some of them were wise, and some of them were foolish." Verily, the latter denomination greatly preponderated; but all of them, whether of the one class or the other,—whether men of letters or men of trade,—whether thinkers or smatterers,—were united in one principle, viz. to traduce a scientific stranger; who, with no great injustice, might claim the praise, given by our great biographer* to another very ill-used child of genius, of being "a man of exalted sentiments, extensive views, and curious observations;—whose remarks on life might have assisted the Statesman, and whose ideas of virtue might have enlightened the Moralist." These zealous persons being thorough partisans, were, of course, thorough despisers of candour, and manifested themselves ever ready to decide upon that which they did not know,—to forego science for satire, and to immolate truth at the altar of spleen or fashion. But here let them rest:—turn we now to Dr. Spurzheim.

It is almost unnecessary to say that our approbation, whether expressed or understood, extended only to his anatomical researches,—researches which have been praised and sanctioned by some of the first anatomical names of the day; and many of which had been previously well known in anatomy, but were powerfully opposed and decried by his foemen, simply because they had been so unfortunate as to be re-stated or confirmed by him. But, as to his physiological opinions, the matter was and is far otherwise: here we must exchange the language of assent for that of apology, and must declare that we consider them a mere hypothesis. Certainly, like all other hypotheses of any pretence or ingenuity, Dr. Spurzheim's can quote a few facts which it explains, or cite a few instances which countenance, or seem to countenance it; yet still, we repeat, it is virtually and radically a hypothesis; and we cannot cease to wonder, that a man of his general ability and acuteness, should suffer himself to be the dupe of his own theory. A dupe he certainly is, for as to will-

---

* *See* Johnson's inimitable Life of Savage.

ful imposture, we consider *that* totally out of the question. Before speaking thus confidently, we have taken care to satisfy ourselves that his conclusions are, for the most part, premature or visionary; because, though we cannot pretend to have groped many sconces, or to have gone deep into the art of scalp-measuring, we have seen enough to be convinced that talent is quite compatible with that sort of shape and formation of head to which our author would deny it; and that, on the other hand, many goodly and promising crania are found, upon trial, most sadly to belie their configuration, being either tenantless quite, or tenanted only by dullness, which is much the same thing.

According to this hypothesis, we hear much about expanded or contracted foreheads as being indicative of ability or the reverse; but though the thing may be sometimes, it is by no means uniformly, true: and we hold that Virgil came nigher philosophical accuracy some eighteen hundred and fifty years ago, when he exposed the failures of physiognomy and craniognomy, in his trite apothegm of "nulla fides *fronti*."

Since we are not reviewing a work on organology, the foregoing observations would be altogether irrelevant did they not serve to explain the reason of our extreme tardiness in laying the outline of Dr. Spurzheim's present treatise before our readers. The fact is, that entertaining the above opinions of his peculiar theory of the functions of the brain, we conceived it would be an ungracious task to analyse a work which (as may be gathered from the title) wholly and fundamentally leans upon that theory; and we anticipated no little trouble in separating "the wheat from the chaff;"—or, in disentangling the valuable pathological and practical facts about insanity, (and there are many such in this book) from the peculiar tissue of hypothesis with which they are entwined. This, nevertheless, is what we shall endeavour to do; and although we have put off labour to the longest day, we are conscious it would be unpardonable in us to suffer another volume to close upon our readers, without duly executing this portion of our duty.

The work is divided into two parts:—1st. Of the derangements of the external functions of the mind; and, 2d. Of the derangements of the internal functions of the mind.

The first part contains two chapters, the one upon disorders of voluntary motion, such as convulsions, epilepsy, catalepsy, palsy; and the other upon diseases of the five senses. The second part likewise contains two chapters, the one upon diseases of the brain, viz. cephalalgia, vertigo and lethargic affections, apoplexy, phrenitis, and hydrocephalus acutus; the other treats of insanity, properly so called, and consists of seven sections, detailing the symptoms, causes, and other particulars relative to this dreadful malady, and its treatment.

The introduction occupies thirteen pages, and is not badly written; only we think the author is scarcely correct, when he insinuates that the present depressed state of our knowledge of insanity is owing to the very small degree of attention given by medical men to the study of the mind in general, and of this disease in particular. We would rather account for the deficiency of our knowledge from the great difficulty of the subject, and our almost absolute ignorance of the physiology of the nervous system. Here physiological must necessarily precede pathological knowledge, for (as Haslam* has justly observed) "whenever the functions of the brain shall be fully understood, and the use of its different parts ascertained, we may then be enabled to judge how far disease attacking any of these parts, may increase, diminish, or otherwise alter its functions."

Now, Dr. S. believes he has ascertained the function of the different cerebral parts; ergo, he concludes, that the latter very desirable pathological knowledge is quite within his reach: but he should have spared awhile his self-gratulations, for his conclusion does not follow. Even the most accurate knowledge of structure is a very different thing from knowledge of function.

Our author unquestionably is correct, in thinking that convulsive and paralytic disorders are not diseases of the muscles, but of those portions of the nervous system in which resides the *moving power* of the affected muscles. In the various remarks which he subjoins on the subjects of spasm, epilepsy, and palsy, there is nothing of any importance. He seems to coincide in the views of these diseases, entertained by Dr. Sanders of Edinburgh, and praises that gentleman for the uncommon attention he gives to dissections of the spinal cord. Our readers may see a summary of the opinions of Dr. Sanders, drawn up by himself, in the January number of this Journal: he

* Observations on Madness, 2d edit. p. 237.

maintains, that in all cases of infantile remittent fever (on which disease we have just presented our readers with an original article) where no morbid appearances can be discovered after death, save fæculent accumulations in the colon, if we inspect the spinal canal, we shall find the spinal marrow, and the nerves issuing out from it, of a deep mahogany colour, evidently from congestion of blood in the minute veins; and that this is the cause of death. On the other hand, in convulsive affections, he alleges, that there is always vascular turgescence at the origin of the nerves of the affected parts; and that the irritation thus caused, excites the symptoms, which are symptoms only, as our author contends, and not the disease. Dr. Spurzheim farther illustrates these opinions in this manner:

"Dr. Parry (Elem. Pathol. vol. i. p. 362) judiciously remarks 'the effect of a blow on the ulnar nerve in the elbow, which produces a tingling in the little finger, shows that a disorder may be almost equally perceived in that part of a nerve which is considerably more distant from its origin than the spot on which the irritation was made. This is, indeed, an illustration of the symptoms of paraplegia, which, though usually situated in the spinal marrow, is chiefly perceived in the limbs.' He adds, 'In sciatica the pain is, by the patient, often referred chiefly to the ramifications of that nerve on the outside of the knee, leg, and ancle.'" P. 29.

These ideas are probably, in many cases, substantially correct; but we cannot see how they will explain cases of convulsions or palsy arising sympathically from worms, loaded bowels, disorders of the chylo-poietic function, or other irritations, external or internal. Cases of the latter sort are recorded by Dr. Hamilton, in his useful book on purgatives; and by Mr. Abernethy, in his excellent Surgical Observations on local Diseases; and, indeed, by almost all writers, causes such as the above are held to be by far the most numerous.

After a few meagre remarks about derangements of the external senses, the author passes at once to the second part, on derangements of the internal functions of the mind. We extract the following passage, as it opens up his views, and is the foundation of much of his subsequent reasoning.

"It is known that the internal operations of the mind are often deranged in general diseases, such as in fevers, inflammations, gout, &c.; and every one admits that delirium, stupor, vertigo, lethargic affections, even apoplexy, depend on the cerebral organization.

But, by our ignorance with respect to the functions of the brain, far the greater number of the deranged manifestations of the mind have not yet been generally considered as disorders of the cerebral organization. I think, however, that, as in the disorders of any other organic part, we always consider at the same time its deranged functions; and in observing the deranged functions, we think of its disturbed organization, our proceeding in regard to the brain ought to be the same. Those who speak of diseases of the mind alone, may speak with the same reason of diseases of the mere vital principle in liver complaints, or in disturbed digestion, or in its idiosyncracies. Such physicians may confine their plan of cure to a moral treatment of the archæus, in cases where a person cannot digest mutton or cauliflower.

"Meanwhile I am obliged, in a certain degree, to render my considerations conformable to the general division of nosography; but the time may come, when the derangements of the mental operations, and the disorders of the brain, will be classed in the same order, and only the different disorders of the brain will be spoken of, as is actually the case with the five senses and their organs; when it will be admitted that the deranged functions of the mind are sympathic or idiopathic affections of the cerebral organs; finally, when it will be believed that what happens in all other bodily parts occurs in the brain, viz. that every perceptible derangement of the organization does not visibly affect the function, and that every disturbance of function is not accompanied with a visible alteration in the organization. Accelerated circulation of blood does not always derange the function of the stomach, nor that of the brain. There are cases on record, where a considerable disorganization of the lungs had not disturbed respiration, and was detected only after death. More details of this kind are mentioned in my work on physiognomy, where I answer the objection, founded on the injuries of the brain, against the proposition that the brain is the organ of the mind." P. 39-41.

This, according to our notions, is sound pathology; and although it is by no means peculiar to, or original with, our author, we cannot deny him the praise of having chosen the right path, and seeing his way clearly before him. He has contributed his strength to overthrow the long-established error of the mind's being liable to diseases of its own, independent of its material receptacle—the body. This error, we believe, first took its rise from the mystical speculations of the Egyptian metaphysicians, who detached the study of the mind from the pursuits of natural philosophers, investigated the agent as independent of organization, and involved the subject in hieroglyphics and mummery, that they might the better enhance the reverence for their supposed knowledge, and extend the sacerdotal office to the treatment of mental alienations as well as other diseases. It was farther rooted, strengthened, and handed down to the present times, by Descartes and the rest of his sect, who long ruled the schools, and gave the tone to the reigning philosophy of the age. It is an error, we think, which has operated more than any other cause, to prevent the establishing of a correct theory and judicious treatment of insanity; but it is an error which has vanished away before the sun of modern physiology; it exists no longer, except in the brains of poets, rhetoricians, and schoolmen.

*Apoplexy.* Dr. S. treats of it briefly under its three divisions into sanguineous, serous, and nervous. To the latter species he refers sudden deaths from excessive emotions of joy, fear, &c. and those cases where persons "sub coitûs actu perierunt." The following paragraph is practical.

"Serous apoplexy is observed in adults under circumstances which, in children, produce hydrocephalus acutus; that is, in individuals with an inflammatory diathesis of the brain, where the great determination of blood to the head produces a serous effusion. In strong individuals, it may appear as phrenitis. In weak persons, its progress is slow, and the modified appearances induce those physicians who consider merely symptoms, and not the nature of the disease, to speak of different diseases. There are, however, acute and chronic inflammations in the brain, as in any other part of the body, and every where the same morbid changes may be observed. Serous effusion is not so unusual in adults as is believed. It is often found in those who die of chronic mania, or who had become idiotic from that disease." P. 47.

*Hydrocephalus Acutus.* Our author gives a very accurate and animated picture of this terrible malady so often fatal to the parent's hope. We regret much that our limits will not permit us to do his delineations any thing like justice.

He is of opinion that this disease can be prevented, but can never be cured: this is only true if the term hydrocephalus is restricted to the second and third stages; if it is applied to the first stage, or that of excitation, (and we think it as applicable to it as the term typhus is to the active as well as to the putrefactive stage of malignant fevers) then, we hold, the disease is not only cureable, but also is very often cured. In the chronic species, we believe he is right, when he says it is in vain to torment the unhappy child with blisters, issues, mercury, digitalis, and so on.

More girls than boys, he thinks, suffer from acute hydrocephalus, and scrofulous, weak, delicate infants, and also stout and strong ones of a fair complexion are subject to it.

Dr. Spurzheim on leading points both of opinion and practice, coincides with Dr. Yates and Dr. Cheyne; but he differs from them as to the frequency of previous abdominal affection as a precursor of hydrocephalus acutus.

"Anatomical dissections have convinced me that, in the greater number of cases, the morbid appearances of the abdomen are secondary symptoms of the affection of the head." P. 57.

The following idea is ingenious:

"In children the brain increases rapidly: hence there is naturally a great determination of blood to the head, and any febrile affection will increase that determination. This is very often the case by dentition, the irritation of which is propagated to the bowels and to the brain. The fifth pair of nerves takes its origin at the same spot with the par vagum, and is in communication with the cerebral parts." P. 59.

We conclude our extracts with some of his valuable practical directions: our author's treatment has our hearty concurrence.

"As some physicians are deceived by greenish evacuations, nausea and vomiting, and prescribe purgatives or emetics; so others are mistaken *(misled)* by the apparent weakness, and employ blisters and stimulating medicines. In children, however, every thing that irritates will increase their naturally great determination of blood to the head. Drastics seldom re-establish the natural secretions, and no liver-complaint is cured by drastics alone: hence, even in cases where the first irritation begins in the abdomen, they ought to be cautiously administered.

"The most inconsistent treatment certainly is when, as it is said, 'every thing is done;'—that is, they draw blood, and at the same time excite the circulation by blisters, emetics, drastic purges, or stimulating medicines. In a healthy and little-irritable child, treated in this manner, fever and head-ache will be produced." P. 62.

Dr. S's plan of cure, and which he asserts to be much more successful than the one reprobated, consists in *tepid* bathing every two hours, venæsection, or leeches to the temples and behind the ears, mild opening glysters, mild diaphoretics, and the avoiding of every stimulus, such as blisters, emetics, drastics, &c.

Even when this disease is cured, impaired mental faculties, and sometimes even permanent idiotism succeed. The author says nothing either for or against the employment of calomel in hydrocephalus acutus. Dr. Blackall, in his late work on dropsies contends, that it is inadmissible where there is coagulable matter in the urine. That gentleman has brought forward new views with regard to the operation of this medicine, and alledges, that it even brings on hydrocephalus after scarlatina. His opinions have had such weight that, to our knowledge, some physicians of very great skill and experience have deviated from the ordinary practice of giving calomel largely in all the diseases of children.

We thus finish our analysis of the preliminary matter of this volume; and here, for the present, we shall stop: the outline of the remainder, which is the most interesting part, as it embraces the main subject of the work, we shall lay before our readers in our succeeding Number.

